# Cannabis use in Tunisian adolescents: Alarming trends from 2013 to 2021: Salsabil Rejaibi

**DOI:** 10.1093/eurpub/ckac131.221

**Published:** 2022-10-25

**Authors:** S Rejaibi, M Zribi, A Silini, M Zid, N Zoghlami, R Mallekh, I Ben Slama, S Ben Youssef, N Ben Salah, H Aounallah-Skhiri

**Affiliations:** 1 Department of Epidemiology, National Institute of Health, Tunis, Tunisia; 2 Medical Faculty of Medicine, Tunis El Manar University, Tunis, Tunisia; 3 Nutrition Surveillance and Epidemiology, SURVEN, Research Laboratory, Tunis, Tunisia; 4 Intensive Care Unit Department, Center for Urgent Medical Assistance, Tunis, Tunisia

## Abstract

**Background:**

Cannabis is the most widely used illicit psychoactive substance worldwide. In Tunisia, the prevalence of cannabis use and its association with other risky behaviours were reported in several publications interesting mainly early adolescence. However, no publications exploring trends based on national epidemiological data are available yet. Our purpose was to determine cannabis prevalence in Tunisian high school adolescents and assess significant trends from 2013 to 2021.

**Methods:**

Pooled data from Mediterranean school surveys on alcohol and other drugs conducted in 2013, 2017, and 2021, were used. Based on three-stage stratification sampling method, first and second grade secondary education students were enrolled. Were not included students enrolled in vocational training centers and out-of-school adolescents. Self-administered standardized questionnaire was used in data collection. We studied weighted lifetime prevalence of cannabis use and chi square test for trend was used for global, by gender and by sector (private/public) trends assessement. STATA software was used for statistical analysis.

**Results:**

A total of 14.723 students were enrolled with sex ratio (M/F) equal to 0.61 and mean age of 16.2±0.8 years. The prevalence of cannabis use increased from 1.4%, to 3.8% then to 7.9% for 2013, 2017 and 2021 respectively. Trend assessement concludes to significant increase in overall cannabis use (p < 10-3). Besides, there was a significant increase in both public and private schools, and among both boys and girls. However, the greatest increase was among male students (3.5% in 2013, 9.2% in 2017 and 16.1% in 2021) (p < 10-3).

**Conclusions:**

Despite the reinforcement of restrictive legislative measures, the prevalence of cannabis use among Tunisian high school adolescents is significantly increasing. Moreover, it’s important to further investigate problematic cannabis use and its effects on adolescents’ physical and mental health.

**Key messages:**

• Trend assessement confirmed the significant increase in lifetime cannabis use in high school adolescents in Tunisia, for both sexes and for both private and public sector.

• This alarming public health issue requires urgent legislation review and close multisectoral collaboration to control supply and demand.

